# Type D Personality Predicts Poor Medication Adherence in Chinese Patients with Type 2 Diabetes Mellitus: A Six-Month Follow-Up Study

**DOI:** 10.1371/journal.pone.0146892

**Published:** 2016-02-19

**Authors:** Xuemei Li, Shengfa Zhang, Huiwen Xu, Xinfeng Tang, Huixuan Zhou, Jiaqi Yuan, Xiaohua Wang, Zhiyong Qu, Fugang Wang, He Zhu, Shuai Guo, Donghua Tian, Weijun Zhang

**Affiliations:** 1 School of Social Development and Public Policy, China Institute of Health, Beijing Normal University, Beijing, China; 2 Clinics of Cadre, Department of Outpatient, General Hospital of the People's Liberation Army (301 Hospital), Beijing, China; 3 Department of Public Health Sciences, University of Rochester School of Medicine & Dentistry, Rochester, NY, United States of America; 4 Department of Psychiatry and Behavioral Sciences, School of Medicine, Duke University Medical Center, Durham, NC, United States of America; Institute of Endocrinology and Metabolism, ISLAMIC REPUBLIC OF IRAN

## Abstract

**Background:**

Type D personality and medication nonadherence have been shown to be associated with poor health outcomes. Type D personality is associated with poor medication adherence in patients with coronary artery disease, myocardial infarction, and heart failure. However, the relationship between type D personality and medication adherence in patients with Type 2 Diabetes Mellitus (T2DM) remains unknown. This study aims to examine whether type D personality was associated with medication adherence in patients with T2DM.

**Design and Settings:**

A follow-up study was conducted in general hospital of the People's Liberation Army in Beijing.

**Methods:**

412 T2DM patients (205 females), who were recruited by circular systematic random sampling, provided demographic and baseline data about medical information and completed measures of Type D personality. Then, 330 patients went on to complete a self-report measure of medication adherence at the sixth month after baseline data collection. *Chi*-square test, *t* tests, and hierarchical multiple regression analyses were conducted, as needed.

**Results:**

Patients with type D personality were significantly more likely to have poor medication adherence (*p<*0.001). Type D personality predicts poor medication adherence before and after controlling for covariates when it was analyzed as a categorical variable. However, the dimensional construct of type D personality was not associated with medication adherence when analyzed as a continuous variable.

**Conclusion:**

Although, as a dimensional construct, type D personality may not reflect the components of the personality associated with poor medication adherence in patients with T2DM, screening for type D personality may help to identify those who are at higher risk of poor medication adherence. Interventions, aiming to improve medication adherence, should be launched for these high-risk patients.

## Introduction

Type D personality describes individuals who simultaneously experience high levels of negative affectivity and social inhibition [1]. Therefore, Type D individuals are thought to experience negative emotions across time and situations, and to inhibit self-expression of these emotions in social interactions due to fears of others’ reactions [2, 3]. Previous study found that Type D personality was associated with a four-fold increased risk of mortality in coronary heart disease (CHD) patients, after controlling the traditional biomedical risk factors [4]. Another follow-up study also indicated that CHD patients with Type D had a four-fold risk of major cardiac events over five years, independent of disease severity [5]. Other studies also found a comparable relationship between Type D and mortality in patients with chronic heart failure (CHF) [6], peripheral arterial disease (PAD) [7], and cardiovascular disease (CVD) [8]. It’s also independently associated with future hemoglobin level, while controlling for renal dysfunction, gender, time since diagnosis, and BMI in in patients with CHF [9]. Another more recent study found that Type D personality was associated with maladaptive health-related behaviors [10], this may represent one mechanism to explain the link between Type D and ill-health.

Given the evidence linking Type D with poor outcome in cardiac populations, several studies have also sought to reveal the potential mechanisms to explain why Type D has a deleterious influence on health outcomes. Previous studies have found a link between Type D and immune activation [11], hyperreactivity of the hypothalamic-pituitary-adrenal axis [12], greater cardiovascular reactivity to stress [13], engagement in fewer health-related behaviours [14] and sub-optimal consultation behaviour [15]. In addtiton, Type D has also been linked to poor adherence to treatment regime in sleep apnoea patients [16]. Three other recent studies indicated that Type D could predict poor medication adherence in patients with heart failure (HF) [17], myocardial infarction (MI) [18], and acute coronary sydrome (ACS) [19].

As of 2014, an estimated 387 million people have diabetes worldwide [20], with type 2 DM making up about 90% of the cases [21]. In China, the overall prevalence of diabetes was estimated to be 11.6% in adult population [22]. Of which, T2DM is the most common type of diabetes, which requires constant attention to diet, blood glucose monitoring, and medication consumption for glycemic control. Poor medication adherence could inhibit the intensification of glucose-regulating [23], and has a strong association with an increased risk of medical complications, e.g. risk of severe cardiovascular diseases and mortality, as well as poor quality of life leading to major health care and economic implications [24, 25]. Therefore, adherence to prescribed medication regimens and persistence with the medication are critical for patients to maintain their health and prevent complications from diabetes, including microvascular and macrovascular disease [26]. It was previously reported that medication adherence in patients with diabetes ranges widely [27], depending on age [28], educational level and socio-economic status [24], depression [29] and other cognitive/physical impairments as well as on regimen complexity [30], dosing frequency [31], fear of hypoglycaemia or of bothersome side effects [32]. Of course, there were also inconsistent findings which reported that gender was a risk factor for medication adherence [33, 34].

Concurrently, illness and medication beliefs are also relevant factors [35]. Lane and colleagues raised the hypothesis that personality traits may offer new insights into variations in glycemic control in patients with T2DM undergoing standard management [36]. Another recent study also emphasized the importance of psychological factors such as personality characteristics (affective temperaments) in medication compliance of patients with diabetes [37]. However, no study to date has examined the link between Type D and medication adherence in patients with T2DM. Therefore, the first aim of this study is to investigate whether Type D personality predicts poor medication adherence in patients T2DM.

The second aim is to determine the utility of Type D as a dimensional construct. Previous study illustrated that Type D may be better considered as a dimensional construct, compared with a categorical construct [38]. Another study also point out that the use of median splits, on both NA and SI, to construct typology may result in some high depressive patients being excluded and some relatively low in depressive symptoms still being placed above the median split [39]. An earlier study also stated the concern about the likelihood of spurious results, when a typological construct like Type D is created from two dichotomized variables [40]. Therefore, it is important to examine the potential statistical problems that may emerge with the dichotomous classification of Type D [39]. Accordingly, this study analyzed the data by using two methods: first, the traditional method of classifying individuals as Type D was used; second, type D personality was analyzed as the dimensional construct, both NA and SI were treated as the continuous variables and performed traditional regression analyses, to test whether the multiplicative term of SI×NA explained the additional variance, after the entry of SI and NA individually.

## Methods

### Ethics Statement

This study was approved by the Ethics Committee of School of Social Development and Public Policy at Beijing Normal University, and the Ethics Committee of First Affiliated Hospital of the General Hospital of PLA. All patients provided written informed consent.

### Participants

The sample procedures were showed in our recently published articles [41]. 412 subjects, who were diagnosed as T2DM according to the diagnostic criteria of the WHO, attended this study from the department of endocrinology at First Affiliated Hospital of the General Hospital of the People's Liberation Army (PLA). All the patients were approached by their attending diabetologist or specialized diabetes nurse. The average age of the subjects was 59.77 years (Standard Deviation, SD = 12.48) with a range of 25–89 years, the patients aged over 60 accounted for 53.6%, and the female made up 50.2%. After giving informed consent, the patients were asked to complete a research questionnaire when they were in hospital and six months later. At baseline, patients completed measures of Type D personality, and provided demographic information. At six months, patients completed a self-report measure of medication adherence. Eventually, 330 (80.1%) of the original 412 participants completed the follow-up questionnaire, at the six-month follow-up, the response rate is 80.1% (330/412). The mean age of the participants at follow-up was 57.24 (SD = 11.45) years, and comprised of 158 (47.9%) females and 172 (52.1%) males. In addition, there were no significant differences between the respondents and non-respondents in terms of gender (χ^2^ = 2.34, *p* = 0.126), HbA1c level at basline (t = -0.14, *p* = 0.989), and Type D personality (χ^2^ = 0.404, *p* = 0.525). However, the respondents were younger than the non-respondents (t = -9.04, *p*<0.001)

### Measurements

#### Demographic information

All eligible subjects were interviewed. The demographic and socioeconomic factors such as age, gender, education level, marriage status, and lifestyle factors were collected at baseline.

#### Type D personality

Type D personality was assessed by the DS14 Chinese version [42], consisting of two seven-item subscales of Negative Affectivity (NA) and Social Inhibition (SI). The NA dimension consists of three lower-order traits (dysphoria, worry, and irritability), and the SI dimension comprises three lower-order traits (discomfort in social interactions, reticence, and social poise) [3]. A five-point Likert scale, ranging from 0 (false) to 4 (true), was used and the cut-off of ≥10 on both subscales indicates Type D personality. The internal reliability were 0.88 and 0.86 for the NA and SI subscales, respectively [3]. For the Chinese version, the Cronbach’s α, for NA and SI, subscales were 0.90 and 0.85, respectively [43]. In this study, the Cronbach’s α were 0.89 and 0.93 for the NA and SI, respectively.

#### Clinical characteristics

At baseline, the clinical outcomes from standard care laboratory tests including HbA1c, Body Mass Index (BMI), the duration of diabetes, and diabetic complications, including hypertension, cardiac disease, diabetic nephropathy, diabetic retinopathy, diabetic peripheral angiopathy and the others, were obtained from the patients’ medical records.

#### Medication adherence

The 8-item Morisky Medication Adherence Scale (MMAS-8), which is a generic self-reported, medication-taking behavior scale, was validated for hypertension [44], and used for T2DM [45, 46], and has been translated into different languages internationally under various settings [46–54]. In this study, MARS-8 Chinese version was used to assess medication adherence in this study.

The MMAS-8 scale adopted a simple and quick scoring algorithm, in the first seven items, negative response for each question was coded as 1, except for the question asking if the patient took their medications yesterday (where a positive response was coded as 1, and a 5-point Likert response for the last item. Item scores are summed to give a score indicating the overall level of adherence. Following the methodology used in the previous study, optimal adherence was defined as having a MMAS-8 score over 6 out of a total of 8 scores [44]. In this study, the Cronbach’s α based on standardized items was 0.84.

### Statistical analyses

To investigate whether Type D can predict medication adherence at Time 2 (T2) after controlling for demographic and clinical variables, two analytic strategies were employed. Firstly, being consistent with Denollet [3], the standard Type D analyses, operationalizing Type D as a categorical variable, were conducted. Secondly, being consistent with standard moderation analyses [55], Type D was operationalized as the interaction between the NA and SI dimensions, the main effects of the NA and SI components were controlled by using the full range of the data. As it suggested that Type D may be a dimensional construct [38], it is also important to treat SI and NA as dimensional constructs and examine the prognostic power of the interaction between the NA and SI traits. Given that one of the assumptions of Type D is that, by definition, its effects are synergistic, i.e. they should only be seen in individuals high in NA and SI, the use of the multiplicative NA×SI term is most appropriate conceptually.

In addition, the univariate analyses were performed to identify the significant variables, before the multiple regression analysis were conducted. In order to determine the adjust-R2 change, all the significant variables were entered into the multiple regression analyses in three steps. In the step 1, the demographic factors, including age, gender, education level, and marriage status were entered into the multiple regression model; then, the clinical factors at baseline including HbA1c value, duration of T2DM, the number of complications, family history of T2DM, BMI, and the behivor factors, including sleeping hours per day and exercise times per week, were also entered into the multiple regression model in the step 2. Finally, Type D was entered into the multiple regression analyses model in the step 3.

## Results

### Subjects characteristics

As shown in Table 1, 92 (43 females) were classified as having a Type D personality (27.9%) using the recommended cut-off point of ≥10 on both NA (M = 10.33, SD = 6.55) and SI (M = 5.44; SD = 5.91) sub-scales. For 330 respondents, 73.94% were married or living with their partners, 10.61% were single (unmarried and divorced), and 15.45% were widowed. Of all the respondents, 11.21% only had a primary education level (≤6 years), 36.97% completed junior middle school/high school (6–12 years), and 51.82% were junior college graduates or above. In addition, retired subjects accounted for 58.48%, as the highest ratio out of all subjects, then followed by occupation (36.36%) and unemployment (5.15%).

**Table 1:** Sample characteristics.

	Total sample (n = 330) n (%)	Type D personality (n = 92) n (%)	Non-Type D personality (n = 238) n (%)	*p* value
**Age**	57.23±11.45	57.78±10.95	57.03±10.95	t = 0.54, *p* = 0.591
**Gender**				
Male	172 (52.12)	43 (46.7)	123 (51.7)	Phi = 0.014, *p* = 0.797
Female	158 (47.88)	49 (53.3)	115 (48.3)	
**Marital status**				
Married	244 (73.94)	59 (64.1)	184 (77.7)	Cramer's V = 0.21, *p* = 0.002
Unmarried	13 (3.94)	8 (8.7)	5 (2.1)	
Divorced	22 (6.67)	4 (4.3)	18 (7.6)	
Widowed	51 (15.45)	21 (22.8)	30 (12.6)	
**Eduaction**				
≥12 years	171 (51.82)	34 (37.0)	137 (57.6)	Cramer's V = 0.21, *p* = 0.001
6~12 years	122 (36.97)	49 (53.3)	73 (30.7)	
≤6 years	37 (11.21)	9 (9.8)	28 (11.8)	
**Employment status**				
Occupation	120 (36.36)	27 (29.3)	93 (39.1)	Cramer's V = 0.091, p = 0.255
unemployment	17 (5.15)	5 (5.4)	12(5.0)	
Retired	193 (58.486)	60 (65.2)	133 (55.9)	

Note: The correlations between Type D personality and gender, marital status, eduaction level, and employment status were analyzed by using the cross table nanlysis, And the Phi and Cramer's V coefficients were utilized, respectively.

### Clinical characteristics

For 330 respondents, the average duration of diabetes was 7.87 years (SD 5.48), with the range of 1–35 years. 80.9% were suffering from diabetes complications, and the top three of complications were hypertension (70.9%), abnormal blood lipid (57.0%), cardiovascular disease (18.8%), and diabetic peripheral neuropathy (18.8%), respectively. In addition, patients with Type D personality presented a longer duration, more complications, and higher BMI, compared with the non-Type D personality patients. Furthermore, No significant difference was observed between patients with or without Type D personality in the variable of HbA1c level at baseline (Table 2).

**Table 2:** The comparison of the clinical outcomes between the patients with or without Type D personality.

	Total (n = 330)	Type D personality (n = 92)	Non-Type D personality (n = 238)	*p* value
Duration	7.87±5.48	9.23±6.73	7.34±4.83	t = 2.83, *p* = 0.003
The number of complications	2.18±1.78	2.47±2.01	2.07±1.67	t = 1.82, *p* = 0.07
BMI	25.00±3.15	25.67±3.05	24.76±3.16	t = 2.37, *p* = 0.009
HbA1c at basline	7.27±1.73	7.37±1.77	7.24±1.72	t = 0.63, *p* = 0.264

### Medication adherence

The mean score for medication adherence was 5.46 (SD = 2.43), ranging from 0 to 8. It was also found that Type D individuals (M = 4.32, SD = 2.50) scored significantly lower than non-Type D individuals (M = 5.90, SD = 2.26) on medication adherence (t = 5.26, *p<*0.001), indicating that Type D individuals reported significantly poorer medication adherence (Table 2).

### Type D personality as a predictor of medication adherence at T2

As shown in Table 3, in the step 1, the inclusion of age, gender, education level, and marriage status did not account for a significant amount of T2 adherence, the total adjust-R^2^ was 0.05, and the combined effect of clinical factors at baseline and behivour factors explained an additional 38% of the variance in the step 2. Comparatively speaking, Type D personality was a significant predictor of medication adherence in the step 3 (β = -1.48, *p*<0.001), by itself, explaining an additional 7% of the variance.

**Table 3:** Hierarchical regression analyses predicting medication adherence at T2.

Step	Variables	β at step (95% CI)	*P* value	Adjust-R^2^
**Step1**	Age	0.018 (-0.006,0.042)	0.136	**0.05**
	**Gender**			
	Male *vs* Female	-1.04 (-1.57, -0.52)	<0.0001	
	**Education**			
	>12 years *vs*≤12 years	0.39 (-0.15,0.94)	0.158	
	**MarriageStatus**			
	Married *vs* Single	-0.30 (-0.89, 0.30)	0.326	
**Step2**	**Age**	0.0002 (-0.02,0.02)	0.983	**0.43**
	**Gender**			
	Male *vs* Female	-0.28 (-0.70, 0.14)	0.201	
	**Education**			
	>12 years *vs*≤12 years	0.39 (-0.04,0.82)	0.074	
	**Marriage status**			
	Married *vs* Single	-0.46 (-0.94, 0.02)	0.062	
	**Clinical factors**			
	Duration	-0.03 (-0.02, 0.07)	0.196	
	Complications	-0.17 (-0.30, -0.04)	0.011	
	Family History	0.42 (-0.00, 0.84)	0.05	
	Baseline HbA1c value	-0.85 (-0.98, -0.74)	<0.0001	
	BMI	-0.06 (-0.12, 0.01)	0.074	
	**Behavior factors**			
	Sleeping hours per day	0.01 (-0.06, 0.07)	0.840	
	Exercise times per week	0.05 (-0.04, 0.14)	0.279	
**Step3**	**Age**	0.007 (-0.03,0.01)	0.7	**0.50**
	**Gender**			
	Male *vs* Female	-0.29 (-0.68, 0.11)	0.150	
	**Education**			
	>12 years *vs*≤12 years	0.16 (-0.24,0.57)	0.429	
	**Marriage status**			
	Married *vs* Single	-0.55 (-1.00, -0.09)	0.017	
	**Clinical factors**			
	Duration	0.05 (0.01, 0.09)	0.014	
	Complications	-0.17 (-0.29, -0.05)	0.007	
	Family History	0.17 (-0.24, 0.57)	0.414	
	Baseline HbA1c value	-0.83 (-0.94, -0.71)	<0.0001	
	BMI	-0.02 (-0.08, 0.04)	0.499	
	**Behivour factors**			
	Sleeping hours per day	-0.01 (-0.08, 0.05)	0.667	
	Exercise times per week	0.06 (-0.02, 0.14)	0.167	
	**Type D personality**^**1**^	-1.48 (-1.93, -1.03)	<0.0001	

Note:

^1^ Type D personality was analyzed as a categorical variable.

Concurrently, the effect of components NA and SI on medication adherence were also examined. As shown in Table 4, when NA and SI, being regarded as the categorical variables, were entered in the regression model (step 1), both NA trait (β = –0.80, *p* = 0.007) and SI trait (β = –0.81, *p* = 0.006) were associated with poorer medication adherence. However, when NA, SI, and their interaction term (NA×SI term), together, were entered into model of the multiple regression, the NA trait predicted the poorer medication adherence (β = –0.63, *p* = 0.042), and the NA×SI term seems likely to make only a marginal difference (β = –1.67, *p* = 0.079), furthermore, the total Adjust-R^2^ increased from 50% in step 1 to 50.3% in step 2.

**Table 4:** Type D persoinality components: NA and SI on medication adherence^1^.

Step	Variables	β at step (95% CI)	*P* value	Adjust-R^2^
**Step1**	**Age**	-0.007 (-0.03,0.01)	0.477	**0.50**
	**Gender**			
	Male *vs* Female	-0.37 (-0.78, 0.04)	0.075	
	**Education**			
	>12 years *vs*≤12 years	-0.16 (-0.57,0.25)	0.444	
	**Marriage status**			
	Married *vs* Being single	-0.64 (-1.1,- 0.19)	0.006	
	**Clinical factors**			
	Duration	0.05 (0.01, 0.09)	0.015	
	Complications at basline	-0.19 (-0.31, -0.06)	0.004	
	Family History	0.15 (-0.26, 0.55)	0.476	
	Baseline HbA1c value	-0.78 (-0.90, -0.66)	<0.0001	
	BMI	-0.02 (-0.09, 0.04)	0.390	
	**Behavior factors**			
	Sleeping hours per day	-0.03 (-0.09, 0.03)	0.331	
	Exercise times per week	0.03 (-0.06, 0.11)	0.505	
	**Type D personality construct**			
	Negative affectivity category	-0.80 (-1.37, -0.22)	0.007	
	Social inhibition category	-0.82 (-1.39, -0.23)	0.006	
**Step2**	**Age**	-0.008 (-0.03,0.01)	0.418	**0.503**
	**Gender**			
	Male *vs* Female	-0.37 (-0.78, 0.04)	0.072	
	**Education**			
	>12 years *vs*≤12 years	0.15 (-0.25,0.57)	0.469	
	**Marriage status**			
	Married *vs* Single	-0.59 (-1.1,- 0.14)	0.011	
	**Clinical factors**			
	Duration	0.05 (0.01, 0.09)	0.011	
	Complications at baseline	-0.18 (-0.31, -0.06)	0.005	
	Family History	0.10 (-0.31, 0.50)	0.645	
	Baseline HbA1c value	-0.79 (-0.91, -0.67)	<0.0001	
	BMI	-0.02 (-0.08, 0.04)	0.527	
	**Behavior factors**			
	Sleeping hours per day	-0.03 (-0.09, 0.03)	0.417	
	Exercise times per week	0.04 (-0.05, 0.12)	0.388	
	**Type D personality construct**^**2**^			
	Negative affectivity category	-0.63 (-1.23, -0.02)	0.042	
	Social inhibition category	0.82 (-1.08, 2.38)	0.463	
	NA×SI term	-1.67 (-3.53, 0.19)	0.079	

Note:

^1^Adjusted for age, gender, education, and marriage status.

^2^Negative affectivity and social inhibition were analyzed as categorical variables.

Next, as shown in Table 5, only SI trait was associated with medication adherence (β = –0.09, *p* = 0.003) (step 1), when type D personality dimensions (NA and SI) were analyzed as continuous variables. Furthermore, none of them were associated with medication adherence (step 2) when NA, SI, and their interaction term (NA×SI term) were entered into the multiple regression.

**Table 5:** Negative affectivity, social inhibition, and medication adherence^1^.

Step	Variables	β at step (95% CI)	*P* value	Adjust-R^2^ for step
**Step1**	**Age**	-0.004 (-0.03,0.01)	0.644	**0.49**
	**Gender**			
	Male *vs* Female	-0.34 (-0.75, 0.07)	0.102	
	**Education**			
	>12 years *vs*≤12 years	0.15 (-0.27,0.57)	0.483	
	**Marriage status**			
	Married *vs* Single	-0.67 (-1.11, -0.21)	0.005	
	**Clinical factors**			
	Duration	0.05 (0.007, 0.094)	0.023	
	Complications	-0.17 (-0.3, -0.05)	0.007	
	Family History	0.19 (-0.22, 0.61)	0.354	
	Baseline HbA1c value	-0.79 (-0.91, -0.67)	<0.0001	
	BMI	-0.03 (-0.09, 0.04)	0.364	
	**Behavior factors**			
	Sleeping hours per day	-0.026 (-0.09, 0.04)	0.417	
	Exercise times per week	0.04 (-0.04, 0.13)	0.354	
	**Type D personality dimensions**			
	Negative affectivity	-0.02 (-0.07, 0.04)	0.516	
	Social inhibition	-0.09 (-0.15, 0.3)	0.003	
**Step2**	**Age**	-0.005 (-0.03,0.02)	0.630	**0.50**
	**Gender**			
	Male *vs* Female	-0.35 (-0.76, 0.06)	0.091	
	**Education**			
	>12 years *vs*≤12 years	0.15 (-0.26,0.57)	0.484	
	**Marriage status**			
	Married *vs* Single	-0.65 (-1.11, -0.19)	0.006	
	**Clinical factors**			
	Duration	0.05 (0.008, 0.096)	0.019	
	Complications	-0.17 (-0.3, -0.05)	0.007	
	Family History	0.17 (-0.25, 0.58)	0.434	
	Baseline HbA1c value	-0.80 (-0.91, -0.68)	<0.0001	
	BMI	-0.022 (-0.09, 0.04)	0.481	
	**Behivour factors**			
	Sleeping hours per day	-0.025 (-0.09, 0.04)	0.453	
	Exercise times per week	0.04 (-0.04, 0.13)	0.297	
	**Type D personality dimensions**			
	Negative affectivity	0.04 (-0.07, 0.07)	0.967	
	Social inhibition	-0.11 (-0.17, 0.15)	0.915	
	Negative affectivity × Social inhibition	-1.01 (-0.02, 0.005)	0.283	

Note:

^1^Negative affectivity and social inhibition were analyzed as the continuous variables.

To further investigate the associations between medication adherence and NA/SI trait, the regression analysis between medication adherence and Type D personality were also conducted. When NA and SI were regarded as continuous variables, the results indicated that only NA trait predicted poorer medication adherence (β = –0.07, *p* = 0.039) (step 1 in S1 Table), and SI seems likely to make only a marginal difference (β = –0.006, *p* = 0.096). Similarly, NA predicted poorer medication adherence (β = –0.08, *p* = 0.043) (step 2 in S1 Table), when NA, SI, and their interaction term (NA×SI term), were entered into the model of regression analysis.

## Discussion

In this study, we explored the association between type D personality and medication adherence in patients with T2DM. To our knowledge, this is the first follow-up study to examine the relationship between type D personality and medication adherence in this patient population. However, two conflicting findings were found. That is, Type D personality was associated with medication adherence when type D personality was analyzed as a categorical variable before and after adjusting for demographic, clinical, and behavior variables. However, when analyzed as a dimensional construct (NA, SI, and NA×SI term), type D personality was not associated with medication adherence.

The former findings are consistent with previous studies on the association between Type D personality and medication adherence in patients with heart failure [17], and on the adverse effects of type D personality on self-management behaviors and getting a regular medical examination [14], when type D personality was analyzed as a categorical variable. In fact, recent studies on the relationship between type D personality and self-management behaviors analyzed type D personality as a categorical variable [16, 56, 57]. Specifically, type D personality had an adverse effect on objectively assessed compliance with continuous positive airway pressure (CPAP) in patients with obstructive sleep apnoea syndrome (OSAS) [16]. Similarly, HF patients with type D personality experienced more HF symptoms, including shortness of breath, fatigue, and sleep problems [56], presented the inadequate consultation behavior and were at a six-fold increased risk of reporting impaired health status, compared to those without type D personality [15]. Furthermore, the odds ratio for treatment discontinuation was 6.03 for type D personality adjusted for covariates [57] in patients with sleep-disordered breathing. The findings above suggested that patients with type D personality had more difficulties to maintain self-management behaviors when type D personality was analyzed as a categorical variable.

Contrarily to the former, type D personality was not associated with medication adherence when analyzed as a dimensional construct. Frankly speaking, we do not know how to interpret the reasons why type D personality was associated with medication adherence when type D personality was analyzed as a categorical variable, but not dimensionally, that is, continuous NA and SI measures and their interaction term. At least, the result needs to be considered from only three studies which have examined the relationship between the dimensional construct of type D personality and medication adherence [17–19]. The findings in this study were consistent with two studies which indicated that dimensional type D personality was not independently associated with medication adherence in patients with ACS [19] and HF [17]. However, another study did show that TypeD personality, as measured as a dimensional construct, predicted medication adherence in patients with MI [18]. Previous studies have also discussed the issues regarding the approaches to conceptualization of type D personality, namely a dimensional or categorical construct [38, 58]. A few articles published recently which exhibited the null findings between type D personality and health outcomes or self-care behaviors in patients with heart disease [59–62]; therefore, these issues should be further studied systematically [58]. Findings in our study suggested there may not be a robust relationship between type D personality and medication adherence. In the future, additional researches are needed to examine the relationship between the dimensional construct of type D personality on medication adherence in patients with T2DM.

The details is still worthy to be discussed. Consistent with the earlier two studies [17, 19], our study showed that the dichotomized NA personality trait was independently associated with medication adherence (β = –0.63, *p* = 0.042), rather than SI and their interaction term (step 2 in Table 4). Besides, being different from three previous studies [17–19], we found that SI was independently associated with medication adherence (β = –0.09, *p* = 0.003) (step 1 in Table 5), when NA and SI were analyzed as continuous variables, however, this association vanished when NA, SI, and their interaction term were entered into the regression model (step 2 in Table 5). It is difficult for us to interpret such discrepancies based on the data of this study. The related studies on this issues in patients with T2DM should be further conducted in the future, to explore the underlying mechanism.

Adherence to prescribed medication is critical for patients with T2DM to have better health outcomes [63–66]. However, patients with T2DM often fail to adhere to treatment for numerous reasons including concerns about administration, mode, timing, convenience, adverse events, and cost [67]. The factors contributing to medication non-adherence in patients taking multiple medications are complex. Some associated factors on the medication adherence in patients with diabetes has been described [68], such as socialeconomic factors [69], medication therapy-related factors [70, 71], patient-related factors [72–75], and health system-related factors [76].

There is no study to explore the relationship between type D personality and medication adherence in patients with T2DM. Although, previous studies also emphasized the importance of personality characteristics in medication compliance of patients with diabetes [36, 37]. Several studies revealed that some biological factors, such as cytokines [11, 77] and antioxidant level [78] may explain the relationship between type D personality and cardiac events. However, the underlying relationship between type D personality and poor outcomes are largely unknown, especially in patients with T2DM. Even though, two conflicting findings were found in this study, the association between adverse health outcomes, medication adherence and type D personality should paid more attention in patients with T2DM.

Findings on medication adherence in this study contribute to the growing body of evidence for the involvement Type D personality and self-management in patients with T2DM. As highlighted above, Type D patients may possess inadequate consultation behavior [15]. Theoretically, the SI component of Type D may be crucial for understanding possible subsequent adherence problems, because it depicts an individual who inhibit the self-expression of emotions and behaviors in social interaction [3]. In this study, when NA and SI, as continuous variable, were entered into regression model, only SI predicts the poorer medication adherence, rather than NA (step 1 in Table 5). Although, this effect vanished, when NA and SI interaction term was also entered into the regression model, the importance of SI component could not be ignored, because it may be particularly significant during the social interaction between patients and their physicians.

## Conclusions

In conclusion, this is the first follow-up study to identify a predictive association between Type D personality and medication adherence in patients with T2DM. The findings contribute to a growing body of literature exploring the possible mechanisms between Type D and poor health outcomes in patients with T2DM, although these findings were inconsistent with each other. Thus, screening for type D personality, in patients with T2DM, may help to further investigate the relationship between type D personality and poor medication adherence.

## Limitations

Some limitations should be acknowledged here. First, current study relies on self-report measurement of Type D personality and medication adherence. Social desirability may influence the measurement of medication adherence and may also result in over-estimate of their adherence rates. Future studies should be conducted to examine the relationship between Type D and more objective measurement of adherence. Second, this study was limited by the small sample size and, therefore, we only can adjust some potential confounders that might have an impact on medication adherence in the multiple regression models. Future studies should include a larger sample so that the complex dynamics between type D personality and medication adherence could be better elucidated.

## Supporting Information

S1 Data The data for this manuscript. (DTA)

S1 Table The associations between NA, SI, and medication adherence. (DOCX)

S1 Text The meaning of variables of the data on this manuscript. (DOCX)
