# Supplementary material for: Type D Personality Predicts Poor Medication Adherence in Chinese Patients with Type 2 Diabetes Mellitus: A Six-Month Follow-Up Study
Source: PLoS One. 2016 Feb 19;11(2):e0146892. doi: 10.1371/journal.pone.0146892 (PMC4760773; doi:10.1371/journal.pone.0146892)
Supplement: S1 Table — (DOCX) [file pone.0146892.s002.docx]

| S1 table: The associations between NA, SI, and medication adherence^1^ | | | | |
| --- | --- | --- | --- | --- |
| **Step** | **Variables** | **β at step (95% CI)** | *P* value | **Adjust-R^2^** |
| **Step1** | Negative affectivity | -0.07 (-0.14, 0.004) | 0.039 | **0.11** |
|  | Social inhibition | -0.06 (-0.14, 0.01) | 0.096 |  |
| **Step2** | Negative affectivity | -0.08 (-0.16, 0.002) | 0.043 | **0.104** |
|  | Social inhibition | -0.12 (-0.32, 0.09) | 0.274 |  |
|  | NA × SI term | 0.003 (-0.01, 0.02) | 0.599 |  |

Note: 1 NA and SI were regarded as continuous variables
