# Supplementary material for: Type D Personality Predicts Poor Medication Adherence in Chinese Patients with Type 2 Diabetes Mellitus: A Six-Month Follow-Up Study
Source: PLoS One. 2016 Feb 19;11(2):e0146892. doi: 10.1371/journal.pone.0146892 (PMC4760773; doi:10.1371/journal.pone.0146892)
Supplement: S1 Text — (DOCX) [file pone.0146892.s003.docx]

**S1 text: The meaning of variables of the data on this manuscript**

a11:annual expense

a12: annual expense for diabetes

a16: relative of diabetic patients with positive family history

b2: [height](http://dict.cn/big5/%28a%20person%27s%29%20height)

b3: weight

b23: smoking

b29: alcohol

d1-d14: SSRS scale

h1-h14:type D personality scale
